# Navigating Lymphomas through BCR Signaling and Double-Hit Insights: Overview

**DOI:** 10.3390/hematolrep16010017

**Published:** 2024-03-21

**Authors:** Antonella Argentiero, Alessandro Andriano, Donatello Marziliano, Vanessa Desantis

**Affiliations:** 1 Istituto Tumori “Giovanni Paolo II”, Istituto di Ricovero e Cura a Carattere Scientifico, 70124 Bari, Italy; 2 Pharmacology Section, Department of Precision and Regenerative Medicine and Ionian Area (DiMePRe-J), University of Bari Aldo Moro Medical School, 70124 Bari, Italy; a.andriano2@studenti.uniba.it (A.A.); vanessa.desantis@uniba.it (V.D.); 3 Unit of Internal Medicine “G. Baccelli”, Department of Precision and Regenerative Medicine and Ionian Area (DiMePRe-J), University of Bari Aldo Moro Medical School, 70124 Bari, Italy; donatello.marziliano@uniba.it

**Keywords:** non-Hodgkin’s lymphomas, BCR signaling, double-hit lymphoma

## Abstract

Non-Hodgkin’s lymphomas (NHLs) are a heterogeneous group of lymphoproliferative disorders originating from B, T, or NK lymphocytes. They represent approximately 4–5% of new cancer cases and are classified according to the revised WHO system based on cell lineage, morphology, immunophenotype, and genetics. Diagnosis requires adequate biopsy material, though integrated approaches are used for leukemic presentations. Molecular profiling is improving classification and identifying prognostic markers. Indolent NHLs, such as follicular lymphoma and marginal zone lymphoma, typically pursue a non-aggressive clinical course with long survival. Aggressive diffuse large B-cell lymphoma (DLBCL) is the most common subtype. Recent studies have elucidated pathogenic mechanisms like MYC translocations and BCR pathway mutations. “Double hit” lymphomas with MYC and BCL2/BCL6 alterations confer a poor prognosis. Treatment approaches are evolving, with chemoimmunotherapy remaining standard for many indolent cases while intensified regimens and targeted agents show promise for refractory or high-risk aggressive disease. Continued elucidation of the genetic and microenvironmental underpinnings of lymphomagenesis is critical for developing personalized therapeutic strategies.

## 1. Introduction

Non-Hodgkin’s lymphomas (NHL) are clonal lymphoproliferative diseases originating from B lymphocytes (80–85% of cases), T lymphocytes (15–20%), or natural killer (NK) lymphocytes, which are rare. NHLs represent 4–5% of all new cancer cases in the male and female population and are the ninth leading cause of cancer death in men and the sixth in women [1]. The classification of NHLs is currently based on the criteria proposed by the World Health Organization (WHO), initially modeled on the 1994 REAL classification. NHLs are first identified based on the cell of origin (B, T, or NK lymphocyte) and then on morphological, immunophenotypic, genetic, and molecular criteria integrated with clinical presentation features [2]. In all cases, NHL diagnosis must be based on an examination of adequate biopsy material. An incisional or excisional biopsy is always recommended: since the WHO classification is based on morphological and immunohistochemical criteria, cytological examination of a fine needle aspirate cannot be considered a first-choice method for NHL diagnosis. However, in the case of leukemic lymphomas, which do not present adenopathic localizations easily subjected to biopsy, an integrated diagnostic approach, including morphological examination of the peripheral blood smear and bone marrow aspirate, immunophenotypic characterization of circulating lymphocytes in flow cytometry, and histological and immunohistochemical evaluation of the bone marrow biopsy by expert hematopathologists, allows a diagnosis of virtual certainty in almost all cases. Increasing importance is also acquired by cytogenetic and molecular in-depth analyses for the identification of characterization profiles with prognostic and predictive characteristics usable in therapeutic decisions [3] (see Table 1).

## 2. Indolent B-Cell Lymphomas

These are a heterogeneous group of NHLs characterized by a non-aggressive course, which generally allows long survival even when the disease is not eradicated. Indolent lymphomas represent about half of all NHLs. Follicular lymphoma represents about half of all cases of indolent NHLs. The next most frequent are small lymphocytic lymphomas and marginal zone lymphomas, among which MALT lymphomas of the mucosa-associated lymphoid tissue are the most common [4] (Table 2).

Over the last few years, the incidence of indolent NHLs has increased, albeit to a lesser extent than that of aggressive lymphomas. Age represents the major risk factor associated with the development of an indolent NHL (the median age at diagnosis is 60 years); furthermore, these neoplasms are more frequent in males (with some exceptions, such as certain forms of MALT lymphoma associated with collagenopathies that preferentially affect females) and in the white race. In an epidemiological study conducted on an English population of 5796 patients diagnosed with NHL (years 2004–2012), the frequency of follicular lymphoma was 15.9%, with a significantly lower median age at diagnosis in males (63.1 years) than in females (66.3 years) [5]. A retrospective study conducted on the Swedish population also showed that men are younger at diagnosis (64 vs. 66 years, respectively) [6]. The incidence of primary gastric MALT lymphoma, whose close association with Helicobacter pylori (HP) infection is known, is particularly high in some areas of Northern Italy, probably due to the higher diffusion in these areas of HP infection [7]. Among other infectious agents, HCV plays an important pathogenic role in the onset of indolent lymphoproliferative disorders: meta-analyses report a HCV-positivity prevalence clearly higher in NHL patients (overall risk of 5.7) compared to controls, both in areas endemic for HCV and in those with low prevalence of this infection [8]. This hypothesis is supported by the efficacy of antiviral therapy with alpha interferon and ribavirin observed in some cases of marginal zone lymphoma. Recently, the association between ocular adnexal marginal zone lymphoma and Chlamydia spp infection has been documented. In this case, too, antibiotic therapy (doxycycline) is able to achieve lymphoma remission in approximately 50% of patients who had their lymphoma successfully eradicated [9].

### 2.1. Molecular Genetics

Indolent B-cell lymphomas are partially classified based on the presumed normal counterpart of the neoplastic lymphocyte: while the spectrum of B-cell lymphoproliferative disorders recapitulates the ontogenetic development of normal B lymphocytes, some forms (e.g., hairy cell leukemia) do not clearly correspond to specific differentiation stages. Genetic and molecular investigations often highlight recurrent anomalies and characteristics that better define the stage of maturation and deregulation of the neoplastic cell.

Normal B-lymphocyte precursors undergo rearrangement of surface immunoglobulins (IgM, IgD), thus becoming naive B lymphocytes (CD5+) that circulate in the peripheral blood and are found in primary lymphoid follicles of lymphoid organs and in the mantle zone: most cases of mantle cell lymphoma correspond to CD5+ naive B cells. After encountering the antigen, B lymphocytes proliferate and mature in the context of the germinal center of lymphoid follicles, transforming into centroblasts. These elements express CD10 and BCL6, markers frequently expressed by aggressive B-cell lymphomas presumed to originate from them. Centroblasts undergo intense proliferative activity, developing somatic hypermutations of surface immunoglobulin chains. In the germinal center context, the interaction between centroblasts and T lymphocytes and between centroblasts and dendritic follicular cells determines the processes of negative and positive selection that lead to the maturation of cells to the centrocyte stage, the switching off of BCL6 and the expression of the anti-apoptotic molecule BCL2. Follicular lymphomas derive from B cells of the germinal center (CD10+) that have lost apoptotic control mechanisms, in most cases due to the chromosomal rearrangement t(14;18) that leads to BCL2 overexpression. Leaving the germinal center, mature B lymphocytes recirculate in the peripheral blood and are redistributed in the marginal zones of lymph nodes, spleen, and MALT tissues. Nodal, splenic, and MALT marginal zone lymphomas correspond to memory B cells (CD5− CD10−) in the post-germinal center maturation phase.

The most characteristic genetic and molecular alterations found in indolent B-cell lymphomas are, as mentioned, t(14;18) in follicular lymphoma and t(11;18) in MALT lymphomas: this translocation causes the formation of a fusion gene (API2/MALT1) that determines API2 anti-apoptotic protein overexpression and activation of the MALT1-dependent NF-κB signaling cascade [10].

In recent years, it has become clear that many signaling pathways originating from the B-cell receptor (BCR) are involved in the development, survival, and proliferation of normal B lymphocytes: such signals are implicated, both in a tonic manner and through constitutive activation following punctual mutations, in the pathogenesis of indolent and aggressive B-cell lymphomas. Following these observations, numerous targeted drugs against BCR signaling pathway protein kinases have been designed and clinically applied [11]. New DNA sequencing techniques have also made it possible to identify recurrent genetic lesions in distinct subtypes of indolent lymphomas. Lymphoplasmacytic lymphoma/Waldestrom’s macroglobulinemia is associated in approximately 90% of cases with a point mutation (L265P) of the MYD88 gene, a protein of the Toll-like receptor-dependent signaling pathway, whose constitutive activation determines increased cell proliferation by activation direct and indirect (through Bruton’s Tyrosin Kinase) of NF-κB [12]. Hairy cell leukemia is associated with a point mutation (V600E) of the BRAF gene, a tyrosine kinase component of the MAP kinase signaling pathway that controls cell proliferation and differentiation [13]. Both lesions have demonstrated a pathogenetic role in their respective lymphomas. They can be used as a marker in the differential diagnosis with other forms of indolent lymphomas with similar clinical presentation and are molecular targets for new targeted therapies.

Finally, paralleling the knowledge of mature plasma cell neoplasms [14,15], also in NHL, it has been well demonstrated that the non-neoplastic microenvironment in indolent lymphomas is altered and plays a key role in determining response to treatment and survival [16]. Over 50% of the lymphomatous neoplastic mass is, in fact, represented by T cells, normal B cells, dendritic cells, and macrophages. The composition of the microenvironment (percentage of CD68+ macrophages, ratio between CD4/CD8 lymphocytes and regulatory T cells) is therefore crucial in determining heterogeneous clinical trends, as happens in follicular lymphoma, even if the efforts made to translate biological knowledge on the microenvironment into Validated prognostic models have so far given contradictory results [17].

### 2.2. Morphology

Follicular lymphoma represents the most frequently encountered form of indolent NHL. In most cases, the histopathological diagnosis is based on morphological criteria; in some cases, however, the morphological anomalies can appear very subtle, and the diagnostic alternative of lymphadenitis appears possible. Furthermore, it is not always easy to distinguish a follicular lymphoma from other NHLs characterized by nodular presentation (mantle B-cell lymphoma, marginal zone lymphoma). The most common anomalies of the neoplastic follicle include the decrease in morphological heterogeneity (monomorphism) of the lymphoma cells, the clear decrease in the centrofollicular macrophages (with loss of the starry-sky appearance that characterizes the reactive germinal center), the partial disappearance of the areas mantle cells, the loss of “polarization” and the reduction in mitotic activity (and consequently of the proliferative index which can be assessed by immunostaining with Ki-67). The neoplastic lymphocytes of follicular lymphoma characteristically express B-line markers (CD19, CD20, CD79a) and, in most cases, CD10, BCL6, LMO2, and BCL2; they are normally negative for CD5, CD23, and cyclin D1. Quantitative cytological evaluation of the centroblastic/centrocytic components is important to define the histological grade of follicular lymphoma, defined based on the absolute count of centroblasts (CB) present in 10 neoplastic follicles per high-magnification field (CFI). Grade 1 includes 0–5 CB × CFI, grade 2 includes 6–15 CB × CFI, and grade 3 > 15 CB × CFI: a 3a form (with the presence of a residual portion of centrocytes) and a 3b form (absence of centrocytes). The latter condition presents a similar trend to that of aggressive lymphomas and is normally treated as such [10]. Lymphocytic lymphoma represents the histological counterpart of chronic lymphocytic leukemia, from which it is distinguished by its exclusively nodal presentation: histological analysis of the lymph node shows a characteristic monomorphic appearance corresponding to the proliferation of small lymphocytes. Sometimes the neoplastic component is limited to the interfollicular areas, but frequently, the architecture of the lymph node is subverted by the presence of pseudofollicles (the so-called proliferation centers) containing medium- and large-sized cells (prolymphocytes, paraimmunoblasts). The mitotic index is very low. The neoplastic cells show a characteristic immunophenotype (CD5+, CD23+, CD10−, CD43+, weak CD20, weak surface IgM), identical to that found in chronic lymphocytic leukemia [18].

Marginal zone lymphomas (MZL) include forms with splenic, nodal, and extranodal localization (MALT), the latter in turn divided into primary gastric and non-gastric extranodal lymphomas. The typical immunophenotype of MZL is CD5−, CD10−, CD20+, CD23−/+, CD43−/+, Cyclin D1−, BCL2−, CD103−. CD5 negativity is important to rule out B-cell chronic lymphocytic leukemia or mantle cell lymphoma. Molecular and cytogenetic investigations can highlight characteristic chromosomal anomalies with both diagnostic, prognostic, and/or predictive significance. For example, the discovery that an activating mutation of MYD88 is associated with most cases of lymphoplasmacytic lymphoma has made it possible to develop an allele-specific PCR method useful for better understanding this neoplasm, which often presents with clinical and histopathological characteristics which overlap with other forms, such as MZL [19,20]. It should be underlined that molecular genetic tests must always be integrated into clinical, laboratory, and histopathological diagnostics since no molecular alteration in indolent lymphomas is pathognomonic of a single nosological entity [21].

## 3. Aggressive B-Cell Lymphomas

The definition of aggressive lymphoma is not included in the histopathological classifications but reflects a clinical concept associated with different histological types, united by a rapid clinical course and short survival in cases that are not adequately treated or do not respond to treatment. Over the last twenty years, there has been a constant increase in the incidence of lymphomas without significant differences between sexes and age groups. Compared to indolent lymphomas, the increase in the incidence of aggressive forms was greater, partly linked to the increase in patients with immunodeficiency states. Although the etiology of aggressive NHL, like indolent NHL, remains largely unknown, it is known that congenital or acquired immunodeficiency states represent a significant risk factor. Patients with active HIV infection have a risk of developing aggressive NHL one hundred times higher than that of healthy subjects; some locations, such as the central nervous system (CNS), are particularly frequent in subjects with immunodeficiency. Patients undergoing immunosuppressive anti-rejection therapy following solid organ or bone marrow transplantation are also at risk of developing aggressive NHL. The association between viral infections and lymphomagenesis is well documented even in aggressive lymphomas: EBV infection plays a decisive role in the development of highly kinetic lymphomas, e.g., Burkitt’s lymphoma, as demonstrated by the presence of viral genomic sequences integrated into the DNA of the neoplastic cells; the herpes virus (HHV-8) is implicated in the pathogenesis of Kaposi’s sarcoma and two rare lymphoproliferative diseases, multicentric Castleman’s disease and the so-called “primary effusion lymphoma” (PEL), both more frequently found in elderly or immunocompromised [22,23]. The most frequent form of aggressive NHL is diffuse large B-cell lymphoma (DLBCL), which represents approximately 30% of all lymphomas [4]. The denomination DLBCL includes heterogeneous forms in terms of morphology, phenotype, genetic anomalies, prognosis, and clinical characteristics (e.g., T cell/histiocyte rich DLBCL, primary B lymphoma of the mediastinum, primary B lymphoma of the CNS. From an immunohistochemical point of view, DLBCL constantly expresses B-associated antigens and, in approximately 50% of cases, shows overexpression of the anti-apoptotic protein BCL2: this anomaly has a negative prognostic significance due to the higher incidence of relapses as well as the lower response to therapy. Mantle cell lymphoma (MCL) originates from naïve CD5+ B lymphocytes or, as is believed following more recent studies, from B lymphocytes that have indeed encountered the antigen but not in the context of the germinal center and have not then developed somatic hypermutations [24]. The fundamental genetic characteristic of MCL is the t(11;14) translocation, which causes overexpression and hyperactivation of cyclin D1, an important regulator of the cell cycle, easily demonstrable by immunohistochemistry. The majority of MCL cases express the transcription factor SOX11: the rare SOX11-negative cases have a more indolent course, more frequent leukemia transformation, less nodal involvement, and the probability of progression is lower [25]. The blastoid variant of MCL enters differential diagnosis with acute leukemias and other aggressive types of NHL [26].

Burkitt’s lymphoma is characterized by a very aggressive clinical presentation, by a typical histological pattern (“starry sky”) with the proliferation of all the neoplastic elements (Ki-67 equal to 100%), by the expression of B markers (CD19, CD20, CD22, CD79a) associated with the expression of membrane IgM and the frequent presence of chromosomal translocations on chromosome 8q involving the MYC oncogene.

Lymphoblastic lymphoma is a neoplasm derived from B (approximately 10%) or T (approximately 90%) lymphoid precursors, which mainly affects male subjects in the second and third decades of life. Lymphoblastic lymphoma, whose immunophenotypic characteristics are identical to those of lymphoblastic leukemia of T or B derivation, is differentiated by its exclusively nodal involvement, often in the form of a mediastinal mass. In cases where spinal cord involvement is also present, it is best to define the disease as “lymphoblastic lymphoma” when the proportion of spinal cord blasts is <25% and as “lymphoblastic leukemia” in all other cases [1].

### Molecular Genetics

In recent years, numerous pathogenetic mechanisms have been identified, which have contributed to shedding light on the heterogeneity of the presentation of aggressive B lymphomas and, more importantly, have identified molecular lesions as potential targets for new therapeutic agents.

Lymphomas with a germinal center gene profile (GCB, see below) express characteristic markers (CD10, LMO2, and BCL6), and in approximately 20% of them, a somatic mutation of the EZH2 gene has been highlighted, which favors neoplastic proliferation [27]. In over 50% of GCB lymphomas, but only in 14% of non-GCB cases, reduced expression of PTEN was highlighted, the absence or reduced function of which determines constitutive activation of AKT/mTOR [28]. Lymphomas with an activated gene profile (ABC) derive from B cells in an advanced stage of maturation, with genetic characteristics typical of plasmablasts, including the activation of NF-κB [29]: numerous mutations responsible have been identified for such hyperactivation (including mutations of CARD11, SYK, BTK, PI3K, and MYD88). All these mechanisms justify the preferential action of new drugs in diffuse large cell B lymphomas depending on the gene expression profile. A molecular lesion of particular interest has recently been discovered: the inactivation of the acetyltransferases CREBBP and EP300 [30].

This mutation would be associated with the development of DLBCL and, furthermore, with a fraction of follicular lymphomas: following this alteration, activation of BCL6 and inhibition of TP53 are determined mechanisms that contribute to lymphomagenesis. Molecular lesions of acetyltransferases could represent a target for epigenetic therapy. TP53 mutations are found in approximately 20% of patients with DLBCL; both GCB and ABC are associated with a younger age at diagnosis, higher LDH levels, bulky presentation, and high IPI risk. The presence of TP53 mutations is a strong prognostic indicator of worse survival in DLBCL [31,32].

Through gene profiling studies, various molecular anomalies characteristic of MCL have been identified [33]. The most frequent alteration (42–55% of cases) is the mutation of ATM, a DNA damage sentinel protein, typically associated with 11q deletions and other chromosomal anomalies that confer an unfavorable prognosis. CCND1 mutations, resulting in overexpression of cyclin D1, are associated with a higher proliferation index, reduced survival, and resistance to ibrutinib [34,35]. Activating NOTCH1/2 mutations are found in approximately 10% of cases and are associated with a particularly aggressive trend [36]. The metabolic pathway involving PI3K/AKT is dysregulated in many cases: the activation of AKT supports cell proliferation and may represent a therapeutic target for inhibitors of mTOR, a regulator of AKT [37,38]. Furthermore, overexpression of p21 and SPARC has been documented in MCL: lenalidomide directly represses the expression of these oncogenes, and this could explain its documented efficacy even in patients with advanced and refractory disease [39,40].

## 4. B-Cell Receptor

**Figure 1:**
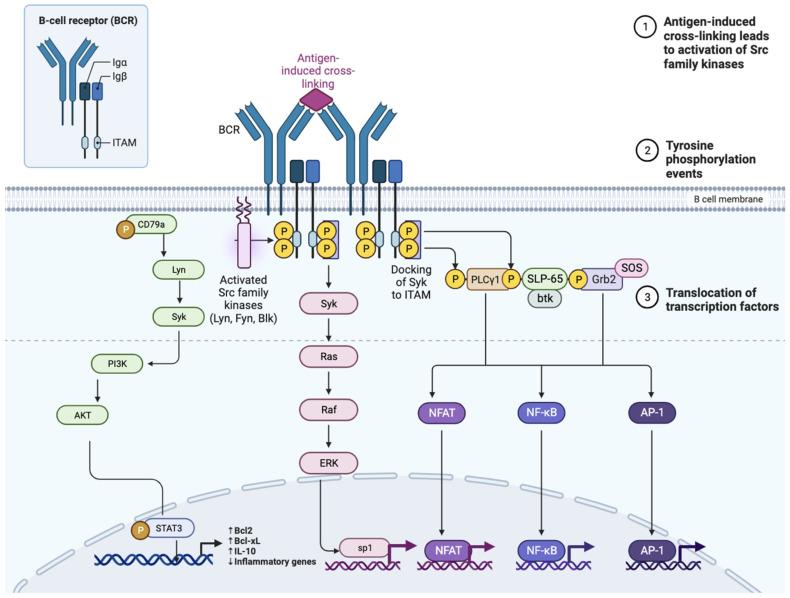
Diagram of the B cell receptor (BCR), the CD19 co-receptor, and the various intermediaries in signal transmission that are activated following the binding of the BCR to the antigen. BLNK, B-cell linker protein; BTK, Bruton tyrosine kinase; CARD11, caspase recruitment domain-containing protein 11; CBM, CARD11–BCL-10–MALT1; CIN85, Cbl-interacting protein of 85 kDa; DAG, diacylglycerol; IKK, inhibitor of NF-κB kinase; IgH, immunoglobulin heavy chain; IgL, immunoglobulin light chain; IP3, inositol trisphosphate; MALT1, mucosa-associated lymphoid tissue lymphoma translocation protein 1; MAPK, mitogen-activated protein kinase; mTOR, mammalian target of rapamycin; NF-κB, nuclear factor-κB; NFAT, nuclear factor of activated T cells; PI3K, phosphoinositide 3-kinase; PIP2, phosphatidylinositol-4,5-bisphosphate; PIP3, phosphatidylinositol-3,4,5-trisphosphate; PKCβ, protein kinase Cβ; PLCγ, phospholipase Cγ; SFK, SRC family kinase.

Many signaling pathways starting from the B-cell receptor (BCR) are involved in the activation, proliferation, and survival of normal and pathological B lymphocytes [41]. Activation of the BCR following antigen binding determines the phosphorylation of CD79A and B and the subsequent recruitment of many intracellular tyrosine kinases, including SYK, LYN, SRC, and BLNK. In turn, these kinases determine the phosphorylation of crucial molecules such as Bruton’s tyrosine kinase (BTK), protein kinase C (PKC), and phosphoinositide 3 kinase (PI3K). This signaling cascade ultimately leads to the activation of ERK, NF-κB, and AKT, implicated in the activation of transcription factors, cell proliferation, and chemotaxis (Figure 1).

Moreover, BCR signaling activates c-Myc, resulting in increased glycolysis and mitochondrial biogenesis. BCR-initiated Ca^2+^ mobilization regulates the metabolic reprogramming of naïve B cells, which is required for their growth and further differentiation [42].

There is evidence that BCR supports tumor cell growth and survival in mantle cell lymphoma MCL, FL, Burkitt’s lymphoma, and marginal zone lymphoma [42]. The idea of targeting BCR-associated kinases emerged as a therapeutic rationale in the last decade, and it led to the development of Bruton’s tyrosine kinase (BTK), spleen tyrosine kinase (SYK), or phosphatidylinositol 3 kinase (PI3K) inhibitors. Targeting BCR signaling with oral kinase inhibitors has changed the treatment landscape in MCL, but this approach seems to be limited by primary or secondary resistance in aggressive B-cell lymphoma, such as diffuse large B-cell lymphoma (DLBCL) [43]. Importantly, in DLBCL, the type of BCR signaling (antigen-driven vs. tonic) reflects gene expression profiling-based cell-of-origin classification into the ABC-DLBCL subtype and germinal center B cell-like GCB-DLBCL subtype, respectively. Moreover, in DLBCL, the role of CD79A and CD79B mutations between different lymphoma subtypes is well established. Mutations in CD79A and CD79B are found in up to 30% of ABC DLBCL cases and only in 3% of GCB DLBCL tumors. CD79B mutations augment BCR surface levels via reduction in BCR endocytosis, preventing BCR binding to clathrin-coated pits [42]; moreover, they increase BCR signaling as a result of failure to properly activate Lyn (a Src-family tyrosine kinase triggering negative feedback loop-based inhibition of BCR signaling). Mutated ITAMs of CD79B promote BCR clustering in ABC DLBCL as a consequence of BCR-antigen binding. The CD79B gene is frequently affected by recurrent splice site mutations in DLBCL that frequently lead to intron 4 retention with premature termination of CD79B translation, resulting in BCR overexpression and enhancement of NF-κB and AKT signaling [44]. On the other hand, phosphorylation of ITAM Y188 of wild CD79A is essential for tonic BCR signal mediation in GCB DLBCL [45].

In DLBCL, the use of tyrosine kinase inhibitors in combination with chemo-immunotherapeutic regimens, such as R-CHOP, showed relevant toxicity. In this sense, a combination of ibrutinib with rituximab and lenalidomide in previously untreated non-GCB cases is showing prolonged responses with some complete responses, suggesting that these new regimens may have a role in frontline therapy, but these results are yet to be confirmed. The most recent trials are combining PI3Ki or BTKi with secondary agents with a distinct mechanism of action, such as the BCL-2 antagonist venetoclax, the cyclin-dependent kinase (CDK)4/6 inhibitor (palbociclib), or together (ibrutinib + umbralisib or ibrutinib + copanlisib), in order to enhance the blockade of BCR signaling. To conclude, BTK and PI3K, in combination with CAR-T and immune checkpoint blockade, may represent significant advances in terms of precision medicine [43].

## 5. Double Hit Lymphomas

The WHO 2008 classification identified a category of aggressive non-Hodgkin’s lymphomas, defined as unclassifiable lymphomas with intermediate characteristics between diffuse large B-cell lymphoma and Burkitt’s lymphoma (hence BL-INT). In many of these lymphomas (up to 80% in some case series), the rearrangement of MYC together with that of BCL2 and/or BCL6 has been identified, a condition defined as “double-hit” (DH) or “triple-hit” (TH) lymphomas depending on the number of pathogenetically relevant lesions identified. Not all BL-INT are “double-hit” and vice versa; there are “double-hit” lymphomas with typical histopathological features of diffuse large B-cell lymphoma and even “double-hit” mantle cell lymphoma, lymphoblastic lymphoma, and follicular lymphoma cases. The topic is controversial, which also reflects the heterogeneity of published case series and makes it difficult to interpret the results of the (few) available clinical studies, as well illustrated in a recent review [46]. The strict definition of “DH lymphomas” applies to cases where a translocation involving the MYC oncogene (locus 8q24) is genetically identified together with the t(14;18)(q32;q21) translocation involving BCL2. There are rare cases where the translocation of MYC and BCL6 is present in the absence of BCL2 involvement (also defined as DH) and equally rare cases where all three translocations are present, a condition defined as “TH lymphomas” [47].

In many papers concerning this category of lymphomas, the cytogenetic evidence of the above-described translocations has not been reported, but only the immunohistochemical overexpression of MYC, BCL2, and/or BCL6. Such expression is not always the consequence of a chromosomal translocation, so the most experienced hematopathologists now agree to define DH or TH only cases demonstrated by cytogenetic testing, reserving the definition of “double-expressor” (DE) lymphomas for the others. The method of choice for identifying MYC, BCL2, and BCL6 rearrangements is FISH. There are two complementary techniques to individually identify translocations of interest. A break-apart probe consists of two fluorescent markers directed toward two regions of the same chromosome upstream and downstream of the known breakpoint, corresponding to the gene of interest; therefore, in the unrearranged normal cell, there will be a fusion signal given by the overlap of the two markers, while in the cell where the break and consequent rearrangement occurred the two markers will be spaced, giving rise to two distinct signals. A dual fusion probe is instead formed by two markers directed toward regions of different chromosomes, known to be the object of the rearrangement. In this case, the normal condition will be the presence of two separate fluorescent signals, and the pathological condition will be the presence of a single fusion signal. In a recent study, MYC rearrangement was evidenced in 38% of cases of DLBCL with the break-apart probe, in 10% of cases with the dual fusion probe, and in 51% of cases using both methodologies concurrently [48]. The diagnosis of DH or TH lymphoma therefore requires the use of FISH for all three genes of interest, which would not be feasible for time and cost reasons in the totality of patients with a new diagnosis of DLBCL. A reasonable approach to identify most DH/TH lymphomas is to analyze with FISH only the lymphomas that have the highest likelihood of presenting the translocations, namely all cases of BL-INT and DLBCLs with high proliferative kinetics (Ki-67 > 80%) and immunohistochemical overexpression of MYC (>40%) and BCL2 (>50%) [49].

Patients with DH lymphoma are generally elderly, and the median age at diagnosis is in the seventh decade of life [47]. The clinical presentation is usually aggressive, with advanced-stage disease, increased LDH, and frequent and extensive extranodal involvement. From a biological point of view, isolated MYC overexpression determines increased cell proliferation but, at the same time, activates proapoptotic signaling pathways through BIM/BAX that balance and mitigate the proliferative effect. In the presence of genetic alterations of BCL2 and BCL6, which inactivate the normal apoptotic pathways of the cell, MYC overexpression results in a much more uncontrolled proliferative drive: this explains why DH lymphomas present greater biological aggressiveness and poor response to conventional treatments [50]. Most studies agree in recognizing that patients with DH lymphoma have lower survival than other DLBCL, while results are still controversial on the prognostic role of the isolated MYC translocation or immunohistochemical overexpression alone of MYC, BCL2, and/or BCL6 (DE lymphomas). A recent analysis on a large retrospective case series of 311 DH lymphoma patients identified four risk factors predictive of survival (Ann Arbor stage 3–4, WBC > 10^3^/uL, LDH > 3 times upper normal limits, central nervous system involvement): patients with 0, 1 or >1 risk factor have a 2-year survival of 91%, 59%, and 41% respectively [51]. Most of the available data on DH lymphoma treatment are based on case series and retrospective observations. One of the first studies [52] analyzed a case series of 303 patients with DLBCL treated with R-CHOP: 14% of patients presented MYC rearrangement, and most of them were defined as DH. Two-year survival was significantly lower in patients with MYC rearrangement/DH (35%) compared to patients with unrearranged MYC (61%). Similar results have been reported in other studies [53,54], while a prospective comparative study between R-CHOP-14 and R-CHOP-21 in patients with DLBCL aged over 60 years [55] did not identify the DH condition as an unfavorable parameter even if a lower survival trend is confirmed (2-year OS of DH patients 63% vs. non-DH 85%, *p* = 0.06).

Considering the clinical aggressiveness, the poor response to R-CHOP, and the at least partial genetic similarity to Burkitt’s lymphoma, many clinicians have treated DH lymphoma patients with intensified therapy regimens. Again, available data refer to retrospective case series with inclusion criteria that are not always homogeneous, but some interesting data seem to emerge. An analysis conducted on 106 DH lymphoma patients treated with various chemotherapy regimens showed that the DA-EPOCH-R regimen (dose-adjusted etoposide, prednisone, vincristine, cyclophosphamide, doxorubicin, rituximab) determines higher rates of complete remission compared to R-CHOP (*p* = 0.01) and other intensified chemotherapy regimens such as R-HyperCVAD and R-CODOX-M/IVAC (*p* = 0.07), with better tolerance especially in older patient [56]. In the MD Anderson experience, including 56 patients retrospectively analyzed, the probability of achieving a complete remission with R-CHOP (20%) was significantly lower than with DA-EPOCH-R (68%) or R-HyperCVAD (70%); the EFS of the entire cohort was only 8 months and no benefits on EFS were observed between transplanted or not transplanted patients in CR. Recently, the preliminary results of a prospective study treating patients with DLBCL or BL-INT with MYC rearrangement with six cycles of DA-EPOCH-R have been reported: at a median follow-up of 14 months, PFS is 79%, and OS is 77%. About half of the treated patients were DH (the others presented isolated MYC rearrangement): the PFS of DH patients (87%) is not lower than that of non-DH patients [57]. According to the latest findings, remarkably, DH-BCL-2 patients and TH patients show akin clinical and molecular features, therefore similar prognostic outcomes that set them apart from DH-BCL6 patients, even though the difference becomes remarkable only after an intensive regimen of chemotherapy including R-CHOP, DA-R-EPOCH, R-CODOX-M-IVAC, R-GCVP, as well as ASCT (n = 1) and CAR-T therapy. These results confirm the need to identify DH-BCL6 in clinical settings to better characterize this subtype [58,59,60]. When combining gene expression profiling data with molecular cytogenetics, interesting results come out. MYC rearrangement prevalence seems to double in GCB compared to the ABC subtype. MYC, as the sole rearrangement as well as in combination with BCL6 rearrangement, was observed in both subtypes. In contrast, HGBL-DH with BCL2 and HBGL-TH appear to be almost exclusively GCB phenomena [61]. On the contrary, the DH/TH signature seems unable to fully represent the aggressive subtype, which remains partially unrecognized. However, most of the GCB lymphomas with a DH/TH negative signature are well responsive to R-CHOP therapy [62]. Lastly, it seems that genetic profiling does not impact the outcome of DLBCL, while DHL and DEL status were associated with worse outcomes in the setting of relapsing/refractory disease [63].

GCB-DLBCL derives from a light-zone centroblast that has gained molecular lesions that induce increased proliferation, the ability to extra-GC migrate, and to “lock in” the GC transcription program. BCL2 translocation is considered one of the earliest genetic events in many B-cell lymphomas. In an identified stage of B-lymphocyte development in the GC light zone, AID and MYC co-expression have been shown, which provides favorable conditions for MYC translocations to occur. One of the most important enzymes that keeps this epigenetic environment in the GC is EZH2. It is found in about 20% of DLBCL and is transcribed in CXCR4-expressing centroblasts. It catalyzes H3K27 trimethylation to silence genes such as IRF4 and PRDM1 (encoding BLIMP-1), which are master regulators of post-GC differentiation. In addition, BCL6 is the key transcriptional repressor that hinders the centroblast from commencing post-GC differentiation. It suppresses the TP53-mediated DNA damage response, permitting genetic recombination and inhibits plasmablast commitment. Moreover, the lymphomagenic effects could be exerted by BCL6 even when not expressed in the centroblast, rather through a transient, stage-specific expression in early hematopoietic cells where it leads to an ABC-like-DLBCL in mice through a durable change in the transcription environment [64]. This could explain the relationship between molecular genetics and cellular morphology.

## 6. Conclusions

In conclusion, NHL represents a diverse group of lymphoid malignancies with variable clinical behaviors and molecular drivers. While R-CHOP therapy has improved outcomes for many common subtypes, heterogeneity persists for those current classifications not fully captured. Emerging insights into genetic lesions, signaling pathways, and the tumor microenvironment from large profiling initiatives are helping to resolve this complexity and identify new targets. Risk-stratified treatment approaches incorporating prognostic markers show promise for optimizing patient care. Further research integrating multi-omics analyses with clinical correlates holds great potential to establish more precise disease definitions supporting individualized management strategies across the spectrum of B- and T-cell lymphomas.

## Figures and Tables

**Table 1:** 2016 WHO Classification of Lymphoid Neoplasms.

**I. Mature (B- and T/NK-Cell) Non-Hodgkin Lymphomas**	
	**1. B-cell lymphomas**
-	-
-	Follicular lymphoma
-	Pedunculated follicular lymphoma
-	Diffuse large B-cell lymphoma
-	Burkitt lymphoma
-	Intravascular large B-cell lymphoma
-	Lymphomas associated with Richter syndrome
-	Diffuse large B-cell lymphoma associated with HIV
-	Plasma cell neoplasm
-	Extranodal marginal zone lymphoma of MALT
-	Nodal marginal zone lymphoma
-	Plasmablastic lymphoma
-	Primary mediastinal large B-cell lymphoma
-	Lymphoma of Waldeyer’s ring
	**2. T/NK-cell lymphomas**
-	Angioimmunoblastic T-cell lymphoma
-	Peripheral T-cell lymphoma
-	T/NK-cell lymphomas
-	Hepatosplenic T-cell lymphoma
-	Anaplastic large cell lymphoma ALK-
**II. Chronic B-cell lymphomas**	
-	Chronic lymphocytic leukemia/small lymphocytic lymphoma
-	B-cell prolymphocytic leukemia
-	Nodal marginal zone lymphoma
-	Lymphoplasmacytic lymphoma
-	Waldenström macroglobulinemia
**III. Mature T- and NK-cell neoplasms**	
	**1. Aggressive lymphomas**
-	Peripheral T-cell lymphoma, not otherwise specified
-	Intravascular T/NK-cell lymphoma
	**2. Indolent lymphomas**
-	T-follicular helper lymphoma
	**3. Well-defined T- and NK-cell neoplasms**
-	T/NK-cell lymphomas
-	Primary cutaneous T-cell lymphoma
	**4. Lymphomas with overlapping features**
-	EBV+ T-cell/NK-cell lymphoproliferative diseases of childhood
**IV. Hodgkin lymphoma**	
**V. Acute lymphoblastic leukemia**	
**VI. Myeloid and myelodysplastic neoplasms**	
**VII. Histiocytic and fibrotic neoplasms**	
**VIII. Plasma cell neoplasms/multiple myeloma**	
**IX. Mast cell neoplasms**	
**X. Neoplasms of histiocytes and dendritic cells**	

**Table 2:** Classification and relative frequencies of indolent B-cell non-Hodgkin lymphomas in adults.

Histologic Type	Frequency (%)
Follicular lymphoma	30–40
Small lymphocytic lymphoma/Chronic lymphocytic leukemia	20–30
Extranodal marginal zone lymphoma	10–15
- MALT lymphoma	5–10
- Other extranodal marginal zone lymphoma	5–10
Splenic marginal zone lymphoma	5–10
Lymphoplasmacytic lymphoma	3–5
Waldenström macroglobulinemia	2–4
Marginal zone lymphoma of small lymphocytes	1–2
Others (<1% each)	5–10
- Nodal marginal zone lymphoma	
- Pedunculated follicular lymphoma	
- Primary bone lymphoma	
- Lennert lymphoma	
Total	100

## Data Availability

No new data were created or analyzed in this study. Data sharing is not applicable to this article.

## References

[1] Jemal A., Siegel R., Ward E., Hao Y., Xu J., Murray T., Thun M.J. (2008). Cancer Statistics, 2008. CA. Cancer J. Clin..

[2] Swerdlow S.H., Campo E., Pileri S.A., Harris N.L., Stein H., Siebert R., Advani R., Ghielmini M., Salles G.A., Zelenetz A.D. (2016). The 2016 Revision of the World Health Organization Classification of Lymphoid Neoplasms. Blood.

[3] Onaindia A., Medeiros L.J., Patel K.P. (2017). Clinical Utility of Recently Identified Diagnostic, Prognostic, and Predictive Molecular Biomarkers in Mature B-Cell Neoplasms. Mod. Pathol. Off. J. U. S. Can. Acad. Pathol. Inc..

[4] Teras L.R., DeSantis C.E., Cerhan J.R., Morton L.M., Jemal A., Flowers C.R. (2016). 2016 US Lymphoid Malignancy Statistics by World Health Organization Subtypes. CA. Cancer J. Clin..

[5] Smith A., Crouch S., Lax S., Li J., Painter D., Howell D., Patmore R., Jack A., Roman E. (2015). Lymphoma Incidence, Survival and Prevalence 2004-2014: Sub-Type Analyses from the UK’s Haematological Malignancy Research Network. Br. J. Cancer.

[6] Junlén H.R., Peterson S., Kimby E., Lockmer S., Lindén O., Nilsson-Ehle H., Erlanson M., Hagberg H., Rådlund A., Hagberg O. (2015). Follicular Lymphoma in Sweden: Nationwide Improved Survival in the Rituximab Era, Particularly in Elderly Women: A Swedish Lymphoma Registry Study. Leukemia.

[7] Doglioni C., Wotherspoon A.C., Moschini A., de Boni M., Isaacson P.G. (1992). High Incidence of Primary Gastric Lymphoma in Northeastern Italy. Lancet.

[8] Matsuo K., Kusano A., Sugumar A., Nakamura S., Tajima K., Mueller N.E. (2004). Effect of Hepatitis C Virus Infection on the Risk of Non-Hodgkin’s Lymphoma: A Meta-Analysis of Epidemiological Studies. Cancer Sci..

[9] Ferreri A.J.M., Govi S., Pasini E., Mappa S., Bertoni F., Zaja F., Montalbán C., Stelitano C., Cabrera M.E., Giordano Resti A. (2012). Chlamydophila Psittaci Eradication with Doxycycline as First-Line Targeted Therapy for Ocular Adnexae Lymphoma: Final Results of an International Phase II Trial. J. Clin. Oncol. Off. J. Am. Soc. Clin. Oncol..

[10] Solimando A.G., Ribatti D., Vacca A., Einsele H. (2016). Targeting B-Cell Non Hodgkin Lymphoma: New and Old Tricks. Leuk. Res..

[11] Pal Singh S., Dammeijer F., Hendriks R.W. (2018). Role of Bruton’s Tyrosine Kinase in B Cells and Malignancies. Mol. Cancer.

[12] Treon S.P., Xu L., Yang G., Zhou Y., Liu X., Cao Y., Sheehy P., Manning R.J., Patterson C.J., Tripsas C. (2012). MYD88 L265P Somatic Mutation in Waldenström’s Macroglobulinemia. N. Engl. J. Med..

[13] Tiacci E., Trifonov V., Schiavoni G., Holmes A., Kern W., Martelli M.P., Pucciarini A., Bigerna B., Pacini R., Wells V.A. (2011). BRAF Mutations in Hairy-Cell Leukemia. N. Engl. J. Med..

[14] Rao L., Giannico D., Leone P., Solimando A.G., Maiorano E., Caporusso C., Duda L., Tamma R., Mallamaci R., Susca N. (2020). HB-EGF-EGFR Signaling in Bone Marrow Endothelial Cells Mediates Angiogenesis Associated with Multiple Myeloma. Cancers.

[15] Di Lernia G., Leone P., Solimando A.G., Buonavoglia A., Saltarella I., Ria R., Ditonno P., Silvestris N., Crudele L., Vacca A. (2020). Bortezomib Treatment Modulates Autophagy in Multiple Myeloma. J. Clin. Med..

[16] Dave S.S., Wright G., Tan B., Rosenwald A., Gascoyne R.D., Chan W.C., Fisher R.I., Braziel R.M., Rimsza L.M., Grogan T.M. (2004). Prediction of Survival in Follicular Lymphoma Based on Molecular Features of Tumor-Infiltrating Immune Cells. N. Engl. J. Med..

[17] Fowler N.H., Cheah C.Y., Gascoyne R.D., Gribben J., Neelapu S.S., Ghia P., Bollard C., Ansell S., Curran M., Wilson W.H. (2016). Role of the Tumor Microenvironment in Mature B-Cell Lymphoid Malignancies. Haematologica.

[18] Tolomeo D., Agostini A., Solimando A.G., Cunsolo C.L., Cimarosto L., Palumbo O., Palumbo P., Carella M., Hernández-Sánchez M., Hernández-Rivas J.M. (2023). A t(4;13)(Q21;Q14) Translocation in B-Cell Chronic Lymphocytic Leukemia Causing Concomitant Homozygous DLEU2/miR15a/miR16-1 and Heterozygous ARHGAP24 Deletions. Cancer Genet..

[19] Xu L., Hunter Z.R., Yang G., Zhou Y., Cao Y., Liu X., Morra E., Trojani A., Greco A., Arcaini L. (2013). MYD88 L265P in Waldenström Macroglobulinemia, Immunoglobulin M Monoclonal Gammopathy, and Other B-Cell Lymphoproliferative Disorders Using Conventional and Quantitative Allele-Specific Polymerase Chain Reaction. Blood.

[20] Sacco A., Desantis V., Celay J., Giustini V., Rigali F., Savino F.D., Cea M., Soncini D., Cagnetta A., Solimando A.G. (2023). Targeting the Immune Microenvironment in Waldenström Macroglobulinemia via Halting the CD40/CD40-Ligand Axis. Blood.

[21] Bogusz A.M., Bagg A. (2016). Genetic Aberrations in Small B-Cell Lymphomas and Leukemias: Molecular Pathology, Clinical Relevance and Therapeutic Targets. Leuk. Lymphoma.

[22] Kaplan L.D. (2013). Human Herpesvirus-8: Kaposi Sarcoma, Multicentric Castleman Disease, and Primary Effusion Lymphoma. Hematol. Am. Soc. Hematol. Educ. Program.

[23] Gloger M., Menzel L., Grau M., Vion A.-C., Anagnostopoulos I., Zapukhlyak M., Gerlach K., Kammertöns T., Hehlgans T., Zschummel M. (2020). Lymphoma Angiogenesis Is Orchestrated by Noncanonical Signaling Pathways. Cancer Res..

[24] Kienle D., Kröber A., Katzenberger T., Ott G., Leupolt E., Barth T.F.E., Möller P., Benner A., Habermann A., Müller-Hermelink H.K. (2003). VH Mutation Status and VDJ Rearrangement Structure in Mantle Cell Lymphoma: Correlation with Genomic Aberrations, Clinical Characteristics, and Outcome. Blood.

[25] Royo C., Navarro A., Clot G., Salaverria I., Giné E., Jares P., Colomer D., Wiestner A., Wilson W.H., Vegliante M.C. (2012). Non-Nodal Type of Mantle Cell Lymphoma Is a Specific Biological and Clinical Subgroup of the Disease. Leukemia.

[26] Rudelius M., Rosenfeldt M.T., Leich E., Rauert-Wunderlich H., Solimando A.G., Beilhack A., Ott G., Rosenwald A. (2018). Inhibition of Focal Adhesion Kinase Overcomes Resistance of Mantle Cell Lymphoma to Ibrutinib in the Bone Marrow Microenvironment. Haematologica.

[27] Béguelin W., Popovic R., Teater M., Jiang Y., Bunting K.L., Rosen M., Shen H., Yang S.N., Wang L., Ezponda T. (2013). EZH2 Is Required for Germinal Center Formation and Somatic EZH2 Mutations Promote Lymphoid Transformation. Cancer Cell.

[28] Pfeifer M., Grau M., Lenze D., Wenzel S.-S., Wolf A., Wollert-Wulf B., Dietze K., Nogai H., Storek B., Madle H. (2013). PTEN Loss Defines a PI3K/AKT Pathway-Dependent Germinal Center Subtype of Diffuse Large B-Cell Lymphoma. Proc. Natl. Acad. Sci. USA.

[29] Davis R.E., Ngo V.N., Lenz G., Tolar P., Young R.M., Romesser P.B., Kohlhammer H., Lamy L., Zhao H., Yang Y. (2010). Chronic Active B-Cell-Receptor Signalling in Diffuse Large B-Cell Lymphoma. Nature.

[30] Pasqualucci L., Dominguez-Sola D., Chiarenza A., Fabbri G., Grunn A., Trifonov V., Kasper L.H., Lerach S., Tang H., Ma J. (2011). Inactivating Mutations of Acetyltransferase Genes in B-Cell Lymphoma. Nature.

[31] Young K.H., Weisenburger D.D., Dave B.J., Smith L., Sanger W., Iqbal J., Campo E., Delabie J., Gascoyne R.D., Ott G. (2007). Mutations in the DNA-Binding Codons of TP53, Which Are Associated with Decreased Expression of TRAILreceptor-2, Predict for Poor Survival in Diffuse Large B-Cell Lymphoma. Blood.

[32] Xu-Monette Z.Y., Wu L., Visco C., Tai Y.C., Tzankov A., Liu W., Montes-Moreno S., Dybkaer K., Chiu A., Orazi A. (2012). Mutational Profile and Prognostic Significance of TP53 in Diffuse Large B-Cell Lymphoma Patients Treated with R-CHOP: Report from an International DLBCL Rituximab-CHOP Consortium Program Study. Blood.

[33] Beà S., Valdés-Mas R., Navarro A., Salaverria I., Martín-Garcia D., Jares P., Giné E., Pinyol M., Royo C., Nadeu F. (2013). Landscape of Somatic Mutations and Clonal Evolution in Mantle Cell Lymphoma. Proc. Natl. Acad. Sci. USA.

[34] Wiestner A., Tehrani M., Chiorazzi M., Wright G., Gibellini F., Nakayama K., Liu H., Rosenwald A., Muller-Hermelink H.K., Ott G. (2007). Point Mutations and Genomic Deletions in CCND1 Create Stable Truncated Cyclin D1 mRNAs That Are Associated with Increased Proliferation Rate and Shorter Survival. Blood.

[35] Mohanty A., Sandoval N., Das M., Pillai R., Chen L., Chen R.W., Amin H.M., Wang M., Marcucci G., Weisenburger D.D. (2016). CCND1 Mutations Increase Protein Stability and Promote Ibrutinib Resistance in Mantle Cell Lymphoma. Oncotarget.

[36] Kridel R., Meissner B., Rogic S., Boyle M., Telenius A., Woolcock B., Gunawardana J., Jenkins C.E., Cochrane C., Ben-Neriah S. (2012). Whole Transcriptome Sequencing Reveals Recurrent NOTCH1 Mutations in Mantle Cell Lymphoma. Blood.

[37] Hofmann W.K., de Vos S., Tsukasaki K., Wachsman W., Pinkus G.S., Said J.W., Koeffler H.P. (2001). Altered Apoptosis Pathways in Mantle Cell Lymphoma Detected by Oligonucleotide Microarray. Blood.

[38] Dal Col J., Zancai P., Terrin L., Guidoboni M., Ponzoni M., Pavan A., Spina M., Bergamin S., Rizzo S., Tirelli U. (2008). Distinct Functional Significance of Akt and mTOR Constitutive Activation in Mantle Cell Lymphoma. Blood.

[39] Zhang L., Kosek J., Schafer P., Bartlett J.B. (2010). Correlation of Tumoricidal Activity of Lenalidomide against Hematologic Tumor Cells with Cyclin D1/D2 Expression and Effect on Tumor-Suppressor Gene Upregulation. J. Clin. Oncol..

[40] Habermann T.M., Lossos I.S., Justice G., Vose J.M., Wiernik P.H., McBride K., Wride K., Ervin-Haynes A., Takeshita K., Pietronigro D. (2009). Lenalidomide Oral Monotherapy Produces a High Response Rate in Patients with Relapsed or Refractory Mantle Cell Lymphoma. Br. J. Haematol..

[41] Young R.M., Staudt L.M. (2013). Targeting Pathological B Cell Receptor Signalling in Lymphoid Malignancies. Nat. Rev. Drug Discov..

[42] Tkachenko A., Kupcova K., Havranek O. (2023). B-Cell Receptor Signaling and Beyond: The Role of Igα (CD79a)/Igβ (CD79b) in Normal and Malignant B Cells. Int. J. Mol. Sci..

[43] Profitós-Pelejà N., Santos J.C., Marín-Niebla A., Roué G., Ribeiro M.L. (2022). Regulation of B-Cell Receptor Signaling and Its Therapeutic Relevance in Aggressive B-Cell Lymphomas. Cancers.

[44] Andrades A., Álvarez-Pérez J.C., Patiño-Mercau J.R., Cuadros M., Baliñas-Gavira C., Medina P.P. (2022). Recurrent Splice Site Mutations Affect Key Diffuse Large B-Cell Lymphoma Genes. Blood.

[45] Havranek O., Xu J., Köhrer S., Wang Z., Becker L., Comer J.M., Henderson J., Ma W., Man Chun Ma J., Westin J.R. (2017). Tonic B-Cell Receptor Signaling in Diffuse Large B-Cell Lymphoma. Blood.

[46] Swerdlow S.H. (2014). Diagnosis of “double Hit” Diffuse Large B-Cell Lymphoma and B-Cell Lymphoma, Unclassifiable, with Features Intermediate between DLBCL and Burkitt Lymphoma: When and How, FISH versus IHC. Hematol. Am. Soc. Hematol. Educ. Program.

[47] Aukema S.M., Siebert R., Schuuring E., van Imhoff G.W., Kluin-Nelemans H.C., Boerma E.-J., Kluin P.M. (2011). Double-Hit B-Cell Lymphomas. Blood.

[48] Tzankov A., Xu-Monette Z.Y., Gerhard M., Visco C., Dirnhofer S., Gisin N., Dybkaer K., Orazi A., Bhagat G., Richards K.L. (2014). Rearrangements of MYC Gene Facilitate Risk Stratification in Diffuse Large B-Cell Lymphoma Patients Treated with Rituximab-CHOP. Mod. Pathol..

[49] Green T.M., Nielsen O., de Stricker K., Xu-Monette Z.Y., Young K.H., Møller M.B. (2012). High Levels of Nuclear MYC Protein Predict the Presence of MYC Rearrangement in Diffuse Large B-Cell Lymphoma. Am. J. Surg. Pathol..

[50] Johnson N.A., Slack G.W., Savage K.J., Connors J.M., Ben-Neriah S., Rogic S., Scott D.W., Tan K.L., Steidl C., Sehn L.H. (2012). Concurrent Expression of MYC and BCL2 in Diffuse Large B-Cell Lymphoma Treated with Rituximab plus Cyclophosphamide, Doxorubicin, Vincristine, and Prednisone. J. Clin. Oncol. Off. J. Am. Soc. Clin. Oncol..

[51] Petrich A.M., Gandhi M., Jovanovic B., Castillo J.J., Rajguru S., Yang D.T., Shah K.A., Whyman J.D., Lansigan F., Hernandez-Ilizaliturri F.J. (2014). Impact of Induction Regimen and Stem Cell Transplantation on Outcomes in Double-Hit Lymphoma: A Multicenter Retrospective Analysis. Blood.

[52] Barrans S., Crouch S., Smith A., Turner K., Owen R., Patmore R., Roman E., Jack A. (2010). Rearrangement of MYC Is Associated with Poor Prognosis in Patients with Diffuse Large B-Cell Lymphoma Treated in the Era of Rituximab. J. Clin. Oncol. Off. J. Am. Soc. Clin. Oncol..

[53] Klapper W., Stoecklein H., Zeynalova S., Ott G., Kosari F., Rosenwald A., Loeffler M., Trümper L., Pfreundschuh M., Siebert R. (2008). Structural Aberrations Affecting the MYC Locus Indicate a Poor Prognosis Independent of Clinical Risk Factors in Diffuse Large B-Cell Lymphomas Treated within Randomized Trials of the German High-Grade Non-Hodgkin’s Lymphoma Study Group (DSHNHL). Leukemia.

[54] Savage K.J., Johnson N.A., Ben-Neriah S., Connors J.M., Sehn L.H., Farinha P., Horsman D.E., Gascoyne R.D. (2009). MYC Gene Rearrangements Are Associated with a Poor Prognosis in Diffuse Large B-Cell Lymphoma Patients Treated with R-CHOP Chemotherapy. Blood.

[55] Cunningham D., Hawkes E.A., Jack A., Qian W., Smith P., Mouncey P., Pocock C., Ardeshna K.M., Radford J.A., McMillan A. (2013). Rituximab plus Cyclophosphamide, Doxorubicin, Vincristine, and Prednisolone in Patients with Newly Diagnosed Diffuse Large B-Cell Non-Hodgkin Lymphoma: A Phase 3 Comparison of Dose Intensification with 14-Day versus 21-Day Cycles. Lancet.

[56] Lu X., Liu Y., Liu R., Liu J., Yan X., Qian L. (2022). Comparison of Chemotherapy Regimens plus Rituximab in Adult Burkitt Lymphoma: A Single-Arm Meta-Analysis. Front. Oncol..

[57] Dunleavy K., Wilson W.H. (2015). Primary Mediastinal B-Cell Lymphoma and Mediastinal Gray Zone Lymphoma: Do They Require a Unique Therapeutic Approach?. Blood.

[58] Khurana A., Mwangi R., Cerhan J.R., Cohen J.B., Chapman-Fredricks J.R., Friedberg J.W., Flowers C.R., Burack R., Lossos I.S., Nastoupil L.J. (2023). Comparing Clinical Characteristics and Outcomes of *MYC* and *BCL6* Double Hit Lymphoma (DHL-*BCL6*) with Other Aggressive B-Cell Lymphomas: Understanding the Impact of New Who and International Consensus Classifications. Blood.

[59] Lue J.K., Luttwak E., Caron P., Boardman A.P., David K.A., Rivas-Delgado A., Epstein-Peterson Z.D., Falchi L., Ghione P., Hamlin P.A. (2023). Clinical Characteristics and Outcomes of Limited Stage High Grade B-Cell Lymphoma with *MYC/BCL2* and/or *BCL6* Rearrangements: A Single Center Experience. Blood.

[60] Shen Y., Cheng S., Xu P., Wang L., Zhao W. (2023). Clinical and Molecular Features of Patients with Double/Triple Hit Large B-Cell Lymphoma. Blood.

[61] Scott D.W., King R.L., Staiger A.M., Ben-Neriah S., Jiang A., Horn H., Mottok A., Farinha P., Slack G.W., Ennishi D. (2018). High-Grade B-Cell Lymphoma with MYC and BCL2 and/or BCL6 Rearrangements with Diffuse Large B-Cell Lymphoma Morphology. Blood.

[62] Ennishi D., Jiang A., Boyle M., Collinge B., Grande B.M., Ben-Neriah S., Rushton C., Tang J., Thomas N., Slack G.W. (2019). Double-Hit Gene Expression Signature Defines a Distinct Subgroup of Germinal Center B-Cell-Like Diffuse Large B-Cell Lymphoma. J. Clin. Oncol..

[63] Desai S.H., Mwangi R., Smith A.N., Maurer M.J., Farooq U., King R.L., Cerhan J.R., Feldman A.L., Habermann T.M., Thompson C.A. (2023). Cell of Origin Is Not Associated with Outcomes of Relapsed or Refractory Diffuse Large B Cell Lymphoma. Hematol. Oncol..

[64] Blombery P.A., Wall M., Seymour J.F. (2015). The Molecular Pathogenesis of B-cell non-Hodgkin Lymphoma. Eur. J. Haematol..

